# Relationship of left ventricular and atrial dimensions with moderate to severe left ventricular diastolic dysfunction (grade II and above)

**DOI:** 10.4314/ahs.v20i4.27

**Published:** 2020-12

**Authors:** Muhammad Hamza, Mishal Fatima, Muhammad Masood, Hafiz Umar Masood, Ghazal Tasleem, Hassaan Ahmed, Maha Nadir, Zubair Satti

**Affiliations:** Rawalpindi Medical College, Cardiology

**Keywords:** Heart failure, diastolic, echocardiography, left ventricle

## Abstract

**Introduction:**

Left ventricular diastolic dysfunction (DD) is an entity in which the ventricle fails to fill up properly due to impaired ventricular relaxation and/or decreased compliance. The diagnosis of diastolic dysfunction is based on a variety of parameters in doppler echocardiograpy. However, some parameters like interventricular septal thickness in diastole (IVSd), posterior wall thickness in diastole (PWd), left ventricular internal end diastolic and systolic diameters (LVIDD and LVISD) along with left atrial diameters (LAD) have yet to be evaluated for the diagnostic workup of DD.

**Methods:**

A case control study was done in the cardiology department from patient records from 2016 to 2018. Patients were diagnosed as diastolic dysfunction grade II and above by doppler echocardiography. IVSd, PWd, LVIDD, LAD, LVISD were obtained through 2-D echocardiography.

**Results:**

Patients with DD had greater LAD, IVSd and PWd and decreased LVIDD and LVISD as compared to control group. Overall, IVSD was the most significant predictor (OR 1.52 95%CI 1.35–1.71) of DD followed by PWd and LAD. Similarly, LAD, IVSd and PWd had higher sensitivity and specificity than LVIDD and LVIDS.

**Conclusion:**

IVSd, LAD and PWd showed significant performance in the diagnosis of diastolic dysfunction and hence can be used as a screening and diagnostic tool in diastolic dysfunction of grade ll and above.

## Introduction

Left ventricular diastolic dysfunction (DD) is a clinical entity in which the ventricle fails to fill up properly due to impaired ventricular relaxation and/or decreased compliance of the ventricles. The most common cause of this is concentric hypertrophy due to arterial hypertension, senility and aortic stenosis but other conditions like restrictive cardiopathy and constrictive pericarditis can also cause diastolic dysfunction. This adaptive response is mainly due to sustained hypertension, aortic stenosis and advanced age. Diastolic dysfunction has similar symptoms as systolic dysfunction like dyspnea, exercise intolerance and edema but it has preserved or slightly decreased ejection fraction^1^. DD is graded into 3 types from mild (grade I), moderate (grade II) and severe (grade III)^2^ The diagnosis of diastolic dysfunction is based on doppler echocardiography and E/A ratio (E is the velocity of blood during early phase of diastole and A is the velocity during atrial phase) is the main diagnostic criteria to grade diastolic dysfunction. The left ventricular geometric indices like interventricular septal thickness in diastole (IVSd), posterior wall thickness in diastole (PWd), left ventricular internal end diastolic and systolic diameters (LVIDD and LVISD) along with left atrial diameters (LAD) have mostly remained in the realm of theoretical description of diastolic dysfunction. Literature review shows that these indices remain neglected in the clinical screening or diagnosis of diastolic dysfunction^3^,^4^.

DD is quite a common disease with an overall 25–30% prevalence in general population and its prevalence increases with age. It is correlated with significant cardiac morbidity and mortality if remained undiagnosed and untreated.^5^ This problem is made even more challenging due to the relative ambiguity and limitations in its diagnostic indices (namely pseudo normalization of E/A in grade II disease)^1^,^2^,^5^. Therefore, multi modal diagnostic criteria involving many indices are encouraged for diagnostic work up of DD.^2^

Left ventricular geometrical indices have been mainly confined to theoretical concept of DD and little work has been done on their application in clinical diagnosis. These indices can easily be assessed using echocardiography and can provide additional tools in the multi modal diagnostic arsenal available for DD.

Many studies have shown the values of left ventricular geometrical indices in DD. IVSd, PWd, LAD and ejection fraction (EF) increases while LVIDD and LVISD decreases in DD^6^,^4^. However, there is a relative dearth of literature that evaluates the diagnostic abilities of these indices. Recently, a novel parameter LAD/LVIDD was proposed as a screening test for DD of grade II and above^6^.

Thus, this study aims to evaluate left ventricular geometrical indices in the screening and diagnosis of DD grade II and above.

## Materials and Methods

A case control study was done in the cardiology department from patient records from 2016 to 2018. Left Atrial Diameters (LAD) were obtained on 2-Dimensional (2-D) Echocardiography in apical views (four chamber view) during end systole. Interventricular septal and posterior wall thicknesses at end-diastole along with left ventricular internal diameters at both end diastole and end systole were obtained on 2-Dimensional (2-D) Echocardiography in parasternal long axis view (4 chamber view).

In patients with normal EF without any evidence of myocardial disease, diastolic dysfunction was diagnosed on 2D and Doppler Echocardiography when all three of these variables were abnormal (Annular septal e' velocity less than 7cm/sec or lateral e' velocity less than 10cm/sec, E/e' ratios greater than 14, LA volume index greater than 34mL/m^2^) as per American society of Echocardiography gudielines^2^. Grade 2 DD was then categorized when E/A ratios were either less than or equal to 0.8 with Mitral E velocities greater than 50 cm/sec or when E/A ratios were between 0.8 and 2.2.

In patients with reduced EF (less than 50%) or patients with preserved EF (greater or equal to 50%) and myocardial disease (e.g. Coronay Artery Disease) diastolic dysfunction grade II was diagnosed when E/A ratios were either less than or equal to 0.8 with Mitral E velocities greater than 50 cm/sec or when E/A ratios were between 0.8 and 2 along with these two abnormal variables (E/e' greater than 14 and LA volume index greater than 34mL/m^2^). Patients having E/A ratio greater or equal to 2 were categorized as Diastolic Dysfunction grade III. Tricuspid regurgitation (TR) velocities were not acquired for the assessment of diastolic dysfunction and therefore an approach consisting of only E/e', E/A, LA volume index and mitral annular velocities was utilized as per American Society of Echocardiographic guidelines 2016 when TR velocities are not available^2^ 178 cases were then randomly selected and compared with 191 controls after careful matching for age, sex, hypertension, diabetes. Patients with rhythm abnormalities and valvular abnormalities were excluded (to facilitate the diagnosis of diastolic dysfunction and to minimize the effect on atrial and ventricular dimensions). Patients with grade l diastolic dysfunction are asymptomatic with a relatively benign course that does not progress in severity. It was excluded from this study due to its negligible effect on ventricular and atrial geometry^7^. Left ventricular geometrical characteristics are size, weight, thickness and volume of the left ventricles that can be obtained using echocardiography. Diastolic dysfunction (DD) is defined as symptoms of systolic dysfunction with preserved or slightly reduced ejection fraction with impaired left ventricular relaxation and decreased compliance.

IVSd, PWd, LAD, LVIDD, LVISD and EF were compared between cases and controls using independent t-test. These indices were correlated with diastolic dysfunction using Spearman correlation. Binary logistic regression (using the above indices) was used to predict diastolic dysfunction. A Receiver Operating Characteristic (ROC) curve was used to check sensitivity and specificity of the above indices. IBM Statistical package for social sciences (SPSS) version 23 was used. A p-vlue of less than 0.05 was considered to be significant.

## Results

The patients with diastolic dysfunction were older and had greater LAD, IVSd and PWd as compared to control group. LVIDD and LVISD were significantly decreased in the diseased group (Table 1).

**Table 1:** Left atrial and ventricular characteristics of the study population

Character	Control group (n=191)	Disease group with DD of grade II and above (n= 178)	Spearman Correlation R	P-value
Gender	M= 81, F=110	M=73, F=105	---	0.8
Age	49.5 ± 18.7	54.8 ±16	0.20	<0.001
LAD (mm)	34.9 ± 7.4	44.6 ± 7	0.50	<0.001
LVIDD (mm)	48.8 ± 7.9	43.4 ± 5.2	-0.30	<0.001
LVISD (mm)	33.4 ± 8.1	26.3 ± 3.9	-0.40	<0.001
IVSd (mm)	9.3 ± 3.0	11.4 ± 2.1	0.46	<0.001
PWd (mm)	9.6 ± 3.1	10.6 ± 1.6	0.30	<0.001
EF (%)	49.1 ± 13.3	56.1 ± 7.3	0.30	<0.001

Values are represented as mean ± SD LAD= Left atrial Diameter, LVIDD= Left ventricular internal end diastolic diameter, LVISD= Left Ventricular internal end systolic diameter, IVSd = Interventriculat septal diameter, PWd= Posterior Wall Diameter, EF= Ejection Fraction

Overall, IVSD was most predictive of DD (OR 1.52, 95% CI 1.35–1.71) as compared to other variables (Table 2).

**Table 2:** Binary logistic Regression for diastolic dysfunction

Variable	Regression Coeffcients (B)	Odds Ratio (ExpB)	95 % Confidence Interval	P-value
IVSd	0.421	1.52	1.35 – 1.71	0.009
PWd	0.185	1.20	1.09 – 1.32	0.012
LAD	0.164	1.18	1.13 – 1.2	<0.001
LVISD	-0.154	0.85	0.82 – 0.89	<0.001
EF	0.064	1.06	1.04 – 1.09	<0.001

LAD= Left atrial Diameter, LVIDD= Left ventricular internal end diastolic diameter, LVISD= Left Ventricular internal end systolic diameter, IVSd = Interventriculat septal diameter, PWd= Posterior Wall Diameter, EF= Ejection Fraction

LAD, IVSd and PWd had higher specificity and sensitivity on ROC curve as compared to LVISD and LVIDD. A new parameter combining LAD, IVSd and PWd was 82% sensitive and 78% specific (Table 3).

**Table 3:** Receiver operating characteristic Curve for diastolic dysfunction

Variable	Area under the curve	P-value	Selected cut off value	Sensitivity	Specificity
LAD	0.82	<0.001	40.5mm	74.2%	79%
IVSd	0.76	<0.001	10.8mm	72%	76%
PWd	0.72	<0.001	10.5mm	71.3%	72%
LVISD	0.25	<0.001	28.5mm	32%	31.9%
LVIDD	0.26	<0.001	44.5mm	37.1%	34%
LAD+IVSd+PWd	0.84	<0.001	58.9 mm	82.6%	74%

LAD= Left atrial Diameter, LVIDD= Left ventricular internal end diastolic diameter, LVISD= Left Ventricular internal end systolic diameter, IVSd = Interventriculat septal diameter, PWd= Posterior Wall Diameter, EF= Ejection Fraction

## Discussion

The ambiguity surrounding diastolic dysfunction stems from its resemblance to systolic dysfunction in clinical presentation and investigations like ECG or chest radiography. Diastolic dysfunction has a chronic long standing course which is asymptomatic in initial stages but can cause significant complications like ischemic heart disease, heart failure or atrial fibrillation in more severe cases. Diastolic dysfunction is common in long standing hypertension, advanced age and diabetes, all of which are also independent risk factors for ischemic heart disease^8^. Therefore, early diagnosis of diastolic dysfunction in these clinical settings can help improve outcomes^9^ by aggressive treatment and management (diuretics, calcium channel blockers, beta blocker(especially nebivolol), angiotensin receptor blocker^10^,^11^. Screening of diastolic dysfunction has also led to improvement in better control of underlying cause (diabetes, hypertension or aortic stenosis) and thus improving outcomes and preventing dire complications^12^.

The diagnosis of diastolic dysfunction is similarly mired in ambiguity and multiple modalities involving various investigations are used in its confirmation. 2-D doppler echocardiography is routinely used to evaluate DD due to its ease of use and cost. Multiple doppler indices like E/A ratio or E/e' ratio are used for this purpose^13^ but these indices also have many limitations as well^2^,^14^. Therefore, new echocardiographic parameters are continuously investigated to be used alongside Doppler indices to enhance diagnostic fidelity^6^. Ventricular and atrial dimensions can therefore provide a new avenue to augment current diagnostic criteria. IVSd, PWd, LAD, LVIDD and LVISD have been shown to be significantly associated with diastolic dysfunction in our study as well as in other studies^7^,^15^. LAD, IVSd and PWd were the most promising dimensions in our study. LAD was 74% sensitive and 79% specific for grade II DD and above. A slightly less sensitivity and specificity for LAD was calculated by another researcher (69% sensitive and 77% specific for grade II and above)^16^ whereas others have demonstrated a slightly better specificity and sensitivity of LAD than the present study^17^. This demonstrates a promising role of LAD as an adjunct to doppler indices for the evaluation of DD. Similarly ventricular dimensions have shown moderate to poor correlation with diastolic dysfunction in this study. The best association was seen with IVSd with r=0.457, AUC 0.762, 72% sensitive and 76% specific. IVSd has been shown to be significantly increased in DD in other studies^4^,^7^, however no study could be found that has evaluated its efficacy in the diagnosis of DD. LVIDD and LVISD is negatively correlated with DD in our study but it is not highly specific or sensitive. Other studies have shown a positive correlation of DD with LVIDD and LVISD^7^,^17^,^18^ which suggests mixed systolic and diastolic dysfunction among patients in these studies. Others have shown a negative correlation^6^. PWd is also significantly associated with DD and is specific and sensitive in the diagnosis of DD as shown by other studies7^17^.

A combination of IVSd, PWd and LAD was 82% sensitive and 74% specific on ROC curve which is higher than each of the dimension alone. This shows that a combined approach utilizing all these dimensions would be helpful in making a correct diagnosis. However, there still is a need to compare and combine these parameters with current doppler indices. This would help ascertain real life benefit of these dimensions in the diagnosis of DD.

## Conclusion

IVSd, LAD and PWd showed significant performance in the diagnosis of diastolic dysfunction and hence can be used as a screening and diagnostic tool in diastolic dysfunction of grade ll and above.
